# General position of Croatian medical biochemistry laboratories on autovalidation: survey of the Working Group for Post-analytics of the Croatian Society of Medical Biochemistry and Laboratory Medicine

**DOI:** 10.11613/BM.2020.020702

**Published:** 2020-04-15

**Authors:** Vladimira Rimac, Anja Jokic, Sonja Podolar, Jelena Vlasic Tanaskovic, Lorena Honovic, Jasna Lenicek Krleza

**Affiliations:** 1 Working Group for Post-analytics, Croatian Society of Medical Biochemistry and Laboratory Medicine, Zagreb, Croatia; 2 Department of Transfusion Medicine and Transplantation Biology, University Hospital Centre Zagreb, Zagreb, Croatia; 3 Department of Medical Biochemistry, Haematology and Coagulation with Cytology, University Hospital for Infectious Diseases “Dr. Fran Mihaljević”, Zagreb, Croatia; 4 Medical Biochemistry Laboratory, General Hospital “Dr. Tomislav Bardek”, Koprivnica, Croatia; 5 Department of Medical Biochemistry and Laboratory Medicine, General Hospital Pula, Pula, Croatia; 6 Department of Laboratory Diagnostics, Children’s Hospital Zagreb, Zagreb, Croatia; 7 Croatian Society of Medical Biochemistry and Laboratory Medicine (CROQALM), Zagreb, Croatia

**Keywords:** postanalytical phase, autovalidation, clinical laboratory, questionnaire

## Abstract

**Introduction:**

Autovalidation (AV) is an algorithm based on predefined rules designed, among others, to automate and standardize the postanalytical phase of laboratory work. The aim of this study was to examine the overall opinion of Croatian medical biochemistry laboratories regarding various aspects of AV.

**Material and methods:**

This retrospective study is an analysis of the responses of a survey about AV comprised of 18 questions, as part of Module 10 (“Postanalytical phase of laboratory testing”) of national External Quality Assessment program, administered by the Croatian Centre for Quality Assessment in Laboratory Medicine. Results were reported as percentages of total number of participants in survey or as proportions of observed data if the overall number of data was <100.

**Results:**

121 laboratories responded to the survey, of which 76% do not use AV, while 11% of laboratories use AV in routine laboratory work. 16/29 laboratories implemented semi-automated AV for general biochemistry (7/29), haematology (5/29), and coagulation (4/29) tests. Analytical measurement ranges, critical values, flags from analysers, interference indices and delta check were the most commonly used rules in the algorithm. 12/29 laboratories performed validation of AV with less than 500 samples (8/29). 7/13 laboratories report the percentage of AV being 20-50%, while 10/13 answered that introduction of AV significantly reduced turnaround time (TAT) (for 20 - 25%), especially for biochemistry tests.

**Conclusions:**

Despite of its numerous benefits (*i.e.* shorter TAT, less manual validation, standardization of the postanalytical phase), only a small number of Croatian laboratories use AV.

## Introduction

Everyday pressures made upon laboratories to produce and release laboratory test results more promptly urged laboratory professionals to develop schemes and procedures, which could maximize the efficiency of the postanalytical phase of laboratory work. Well-thought-out schemes for automated selection and reporting of test results procedures of the postanalytical phase, or autovalidation (AV), contribute to shorter turnaround time (TAT), reduce the need for the laboratory personnel’s manual validation, and diminish error rate (*1*). The purpose and benefit of AV is not just postanalytical phase automation but also better work efficiency in laboratory.

Autovalidation is an algorithm based on predefined rules (*i.e.* quality control results, flags from the analyser, delta check, critical values) designed, among other, to automate and standardize the postanalytical phase, reduce workload of laboratory professionals, and enable them to pay more attention and devote time to the problematic or challenging laboratory test results (*2*). The rules in AV algorithm must be clearly defined, tailored to the patient population in the laboratory. In addition, all rules in algorithm should be equally valuable, and all test results checked through all rules in algorithm. Before implementation of AV in routine work, it is necessary to perform validation of AV, to verify that the rules and criteria in algorithm are properly defined (*3*, *4*).

Recently, the Working Group for Post-analytics of the Croatian Society of Medical Biochemistry and Laboratory Medicine (CSMBLM) published national recommendations “Post-analytical laboratory work: national recommendations from the Working Group for Post-analytics on behalf of the Croatian Society of Medical Biochemistry and Laboratory Medicine”, suggesting and recommending AV as one of the procedures which can contribute to higher efficiency and quality of the postanalytical phase (*3*). This study is the first extensive and more detailed insight into the use of AV among Croatian medical biochemistry laboratories after national recommendations were released. This study was conducted in the attempt to determine the general opinion of Croatian medical biochemistry laboratories about AV: how many laboratories use AV in routine laboratory work, what are the benefits of AV in laboratories where it is used, and the reasons why laboratories do not use AV.

## Materials and methods

### Study design

This retrospective study was designed as a survey on AV conducted by the Working Group for Post-analytics in cooperation with the Croatian Centre for Quality Assessment in Laboratory Medicine (CROQALM). In June 2019, an online questionnaire was distributed to all 195 Croatian medical biochemistry laboratories as part of the national external quality assessment (EQA) scheme, administered by the CROQALM within the CSMBLM. Participation in the survey was voluntary. Laboratories that responded to the survey were included in the study with no exclusion criteria.

### Questionnaire

As part of the EQA scheme, Module 10 entitled „Post-analytical phase of laboratory testing” contained a survey comprised of 18 questions. Survey participants were offered multiple-choice answers for some questions. All questions and answers of the survey are presented in Tables 1-3.

**Table 1:** Questions related to reasons for introducing/not introducing AV in routine laboratory work

**Questio**n	**Answers**	N (%)*
1. What is the type of your institution?	Primary health care (private medical practice and private laboratories)	72 (60%)
	Secondary health care (general, national and special hospitals)	31 (25%)
	Tertiary health care (clinical hospitals and clinical hospital centres)	18 (15%)
2. Do you use automated selection and reporting of test results („autovalidation“- AV) in your laboratory?	Yes	13 (11%)
	In the introduction process	16 (13%)
	No	91 (76%)
	Reasons why AV is not used:	
	a) small number of samples/patients	27/91
	b) old version of the LIS/inability to implement AV in existing LIS	11/91
	c) insufficient financial resources	7/91
	d) no need for AV in laboratory	5/91
	e) insufficient information about AV	10/91
	f) laboratory is in the process of introduction of AV	4/91
	g) problems with IT support	2/91
	h) it is planned to introduce AV in the laboratory	4/91
	g) does not contribute to improving the quality of work	2/91
	i) no answer to the question	19/91
3. Why did you decide to introduce AV in your laboratory?	a) improving accuracy of the test results	3/29
	b) a large number of samples/patients/tests	4/29
	c) quality and reorganization of routine laboratory work	11/29
	d) shortage of laboratory staff	2/29
	e) no answer to the question	9/29
*Results are reported as percentages of total number of participants or as proportions. AV - autovalidation. LIS - laboratory information system. IT - information technology. N - number of laboratories that answered the question.		

**Table 2:** Questions related to the technical creation of the AV algorithm, definition of rules and criteria, and validation of AV

**Question**	**Answers**	**N**
1. Autovalidation algorithm is:	a) a part of LIS used in the laboratory	20/29
	b) laboratory has an independent program for AV	1/29
	c) implemented in the middleware	0/29
	d) no answer to the question	8/29
2. Technically, AV is:	a) semi-automated	16/29
	b) automated - „real-time“	4/29
	c) no answer to the question	9/29
3. AV is implemented:	a) at the sample level	12/29
	b) at the test level (for „real-time“ AV)	8/29
	c) no answer to the question	9/29
4. Which test panel is included in AV algorithm?	All tests performed in the laboratory	8/29
	Emergency tests only	1/29
	Part of the tests performed in the laboratory	11/29
	The test panels included in AV are:	
	a) general biochemistry tests	7/29
	b) haematology tests	5/29
	c) coagulation tests	4/29
	d) special biochemistry tests (*e.g.* tumour markers, immunology tests, therapeutic drug tests)	2/29
	e) urinalysis	2/29
	f) blood gas analysis	1/29
	g) no answer to the question	9/29
5. What criteria were used to set the rules in AV algorithm?	Criteria described in standard operating procedures (SOP) used for manual validation	14/29
	Criteria described in SOP with modification for some tests	3/29
	Criteria from literature/published studies about AV	3/29
	No answer to the question	9/29
6. Rules in your AV algorithm are (multiple choice):	Analytical measurement range	15/29
	Flags from analyser	13/29
	Critical values	15/29
	Delta check	13/29
	Interferences indices (haemolysis, icterus, lipemia)	13/29
	Reference intervals	2/29
7. If delta check is rule in your AV algorithm, how you set the criteria?*	a) by calculating of RCV	3/13
	b) used data from previously published studies	7/13
	c) no answer to the question	3/13
8. Did you performed validation of AV before introducing AV in routine laboratory work?	Yes	12/29
	No	4/29
	No answer to the question	13/29
	If answer is NO, please specify:	
	The validation of AV is in the process	3/4
	No answer to the question	1/4
9. How many samples were included in the validation of AV?	< 500	8/29
	500 - 1000	1/29
	1000 - 2000	4/29
	2000 - 5000	1/29
	5000 - 10,000	1/29
	> 10,000	0/29
	No answer to question	14/29
Results are reported as proportions (only laboratories using AV in routine work and those in validation process). AV - autovalidation. RVC - reference change value. N – number of laboratories that answered the question.		

**Table 3:** Questions on contribution of autovalidation to routine laboratory work

**Question**	**Possible answers**	**N**
1. How long has AV been used in routine work in your laboratory?	Less than one year	5/13
	Between one and two years	4/13
	More than two years	4/13
2. What is the percentage of autovalidated tests results in your laboratory?	< 20%	0/13
	20 - 50%	7/13
	50 - 70%	3/13
	70 - 80%	2/13
	80 - 90%	0/13
	> 90%	1/13
3. In your opinion, has the introduction of AV had a significant impact on improvement of TAT?	No	3/13
	Yes	10/13
4. If the answer on the previous question was YES, in which percentage the TAT was reduced?	< 5%	0/10
	5 - 10%	0/10
	10 - 15%	0/10
	15 - 20%	2/10
	20 - 25%	5/10
	25 - 30%	2/10
	> 30%	1/10
5. If AV is introduced for different test panels, on which panel has the most significant reduction in TAT been observed?	General biochemistry tests	5/13
	Haematology tests	2/13
	Coagulation tests	2/13
	Special biochemistry tests (*i.e.* tumour markers...)	0/13
	Emergency laboratory test	3/13
	No answer to the question	1/13
6. In your opinion, what are the benefits of AV in your laboratory? (multiple choice)	Shortening TAT	5/13
	Quality and reorganization of routine laboratory work	6/13
	Reduction of manual validation of test results	4/13
	Improving accuracy of the test results	5/13
Results are reported as proportions (only laboratories using AV in routine work). AV – Autovalidation. TAT - tournaround time. N – number of laboratories that answered the question.		

The first part of the survey contained general questions about type of medical biochemistry laboratory (primary, secondary or tertiary health care) and the application of AV in routine laboratory work. The second part of the questionnaire contained more specific questions concerning AV and was divided into three sections. Questions in the first section were related to algorithm design and included information about the type of information system that was used for AV implementation (laboratory information system (LIS), middleware or an independent AV software), the technical design of the algorithm (semi-automated or automated AV) and whether AV is performed at the sample or test level.

The second part included questions about rule settings in the algorithm as well as validation of AV. Specifically, which test panel and rules were included in AV algorithm, what criteria were used for setting up the rules, and how validation of AV was performed. The last part of survey included question about contribution of AV to routine laboratory work (percentage of autovalidated test results, impact on improvement of TAT, benefits of AV).

### Data analysis

Data were collected through the SurveyMonkey application and data analysis was performed by counting. Results are reported as percentages of total number of participants in survey or as proportions of observed data if the overall number of data was < 100. Data were archived and processed in Microsoft Excel 2010 program (Microsoft, Redmond, USA).

## Results

One hundred twenty-one laboratories responded to the survey, but did not answer all the questions. Although 76% (91/121) laboratories do not use AV, 13% (16/121) are in process of introducing AV in routine laboratory work. Most of the 91 laboratories that do not use AV in routine work stated that the main reasons are small number of samples or patients, inability to implement AV in the existing LIS and insufficient information about AV (Table 1). Regarding the reasons for AV implementation, the answers were almost equally distributed among four answers offered: improving the accuracy of test results, large number of sample/patients/tests, reorganization of routine laboratory work and shortage of laboratory staff.

Table 2 shows answers about technical creation of the AV algorithm, rule settings and validation of AV. Most laboratories implemented semi-automated AV in the LIS for routine biochemistry tests, haematology and coagulation tests, while 8/29 participants answered that AV was implemented for all test performed in their laboratory. Analytical measurement ranges (AMR), critical values, flags from analysers, interferences indices and delta check were the most commonly used rules in the AV algorithm (Table 2). Delta check criteria were either set as reference change value (RCV) or adapted from previously published studies. Moreover, most laboratories stated that for definition of rules in the AV algorithm they used the same criteria used in the laboratory prior to introduction of AV (*i.e.* criteria described in standard operating procedures used for manual validation of test results).

Participants stated that validation of AV was performed before introduction into routine laboratory work or that implementation of AV was in process and validation will be performed. Mostly, validation was performed using less than 500 patient samples (Table 2).

The questions presented in Table 3 were answered only by laboratories that use AV in routine laboratory work. This group of questions relates to the contribution of AV to routine laboratory work (*e.g*. how long AV has been used in routine work, what is the percentage of autovalidated test results and information about effect of AV on improving TAT). Seven out of thirteen laboratories state that percentage of AV test results is 20 - 50% in their laboratories, while one laboratory state that more than 90% test results are released by AV in their laboratory. Ten out of thirteen participants answered that introduction of AV had a significant impact on reducing TAT, especially for general biochemistry tests. Overall, participants state that benefits of introduction AV in routine laboratory work are reduction of TAT, reduction of manual validation, quality and reorganization of laboratory work and improving accuracy of laboratory test results.

## Discussion

Introduction of AV is attractive to laboratory professionals since it bears many benefits, the most commonly reported in our survey being improvement of accuracy of test results, large number of samples/tests/patients in the laboratory, enhancing quality, reorganization of laboratory work and shortage of laboratory staff. There are various reasons for introducing AV in laboratory and recognizing these advantages, the participants in survey answered that reasons for AV implementation. On the other hand, the most common reasons for not introducing AV were small number of sample/patients, insufficient information about AV, inability to implement AV in the existing LIS and insufficient financial resources. Before introducing AV in routine work, it is necessary to be well-informed about AV and establish communication with information technology (IT) specialists, as their assistance in setting up the algorithm is essential.

The first step in the process of AV implementation in routine work is how it will be technically performed. An algorithm can be implemented in several ways: in the LIS used in laboratory, in the middleware or it can function as an independent program for validation (*5*-*13*). In Croatia „semi-automated” AV is most commonly implemented in LIS, where laboratory staff must initiate AV using manual selection. The main benefit of using „semi-automated” AV is the possibility of process controlling because AV can be stopped at any time, *i.e.* when there are technical problems with analysers (*3*). In automated, real time AV, test results are automatically transferred from LIS to the hospital information system (HIS). Therefore, if the laboratory has LIS-HIS communication, real time AV can be implemented. What is more, it gives the possibility to transit from „semi-automated” to real-time AV.

Once the algorithm is technically set up, decision upon whether all tests or specific test panels will be subject to AV should be made. The results of the survey showed that AV is most commonly applied to routine biochemistry tests, haematology and coagulation tests, and similar results are reported in literature (*2*, *14*-*17*). Some studies reported the use of AV for special biochemistry tests, *i.e.* tumour markers, immunology tests, therapeutic drug tests, urinalysis or blood gas analysis (*5*, *7*, *8*, *18*). In this survey, participants also stated that they have implemented AV for special biochemistry tests and urinalysis, and only one laboratory uses AV for blood gas analysis.

Furthermore, it is necessary to decide which rules will be set in the algorithm, but there are no guidelines that state which rules must be part of an AV algorithm. However, previously published studies report that the most commonly included rules in the AV algorithm are: delta check, AMR, critical values, preanalytical and analytical flags from analysers, interference indices and quality control results (*5*, *11*, *13*-*15*, *19*, *20*). In this survey, participants stated that the most commonly used rules are AMR, critical values, flags from analyser, delta check and interference indices. Only two laboratories indicate that reference intervals are used as an AV stopping rule. These are probably primary care laboratories, because if this rule is set in an algorithm in a hospital laboratory, it would yield low percentage of autovalidated test results.

If the laboratory uses AV for different test panels, each test panel could have a dedicated AV algorithm with specific rules (*e.g.* if it is not necessary to determine interference indices for some laboratory test, it should not be the part of the AV algorithm). One of the most useful rules is delta check, because it indicates a significant change of patient’s clinical condition or a problem with a sample (*e.g*. sample mismatch or mis-identification, or preanalytical errors that were not identified before sample analysis) (*3*). Therefore, it is important to define exactly how the criteria for delta check rule will be defined. Participants of this survey answered that criteria used for delta check were usually obtained by calculation of RCV, and this is consistent with literature data (*2*, *14*, *21*).

When creating AV algorithm, rules should be set according to the criteria used for manual validation, with minimal modifications. Most laboratories included in survey answered that they used the same criteria as the ones used prior to introduction of AV.

Before being introduced in routine laboratory work validation of AV should be performed, and the whole process should be documented (*3*, *4*). Most participants in survey answered that they either performed validation, or validation is planned to be performed before the introduction of AV in routine laboratory work, for those laboratories still not using AV. Most participants answered that validation was performed on less than 500 samples. However, data about the number of samples included in the validation process is inconsistent throughout literature (*2*, *14*, *17*). Since there are no guidelines for the number of samples required for validation, Working Group for Post-analytics of the CSMBLM recommends that each laboratory decides on how many samples will be included in the validation, depending on the number of patients/samples in the laboratory and test panel included in AV (*3*).

There are also different data in the literature on the percentage of autovalidated test results. While some studies report that 60 - 85% test results were autovalidated, others state a huge rate of autovalidated test results, *i.e.* above 95% (*1*, *2*, *5*, *7*, *8*, *14*-*17*). Our results show that in the vast majority of laboratories the percentage of autovalidated test results is between 20 and 50%. The underlying reasons for this could be criteria/rules set in AV algorithm (*e.g.* if reference intervals were set as rule in the hospital laboratory, low percentage of autovalidated test results is expected), the patient population in the laboratory, or the type of institution the laboratory is part of (hospital laboratories have grater variation in test results and mostly pathological results). The rules and criteria in AV algorithm should be tailored to the patient population in the laboratory.

It has been mentioned before that AV contributes to shortening of TAT. Most participants answered that TAT was reduced by 5 - 25%, which is similar to data from literature: decrease of TAT by 16% for global coagulation tests, 19% for STAT tests and 22% for routine orders (*5*, *15*).

This study has several limitations. The survey was self-reported, so participants might not be providing reliable information about the real situation in their laboratories. Also, there was a relatively low response rate on questions about AV, *i.e.* a small number of laboratories in Croatia that use AV is also a limitation because it does not allow accurate interpretation of the survey results. However, the results of this study show the main characteristics of the AV algorithm as well as steps and prerequisites or in its design and implementation. Moreover, the results show how to perform validation of AV, and what is the contribution of AV in routine laboratory work, which could be a useful starting point for laboratories that plan to introduce AV in routine laboratory work.

In conclusion, a large percentage of laboratories in Croatia do not use AV in routine practice despite all benefits that AV has an impact on laboratory work. The most common reasons for this are small number of samples/patients in laboratory, probably because most of the responses received in this study are from the small laboratories in the primary care setting. In laboratories where the AV is used regularly, the stated benefits are reduction of TAT and reduction of manual validation, reorganization and increased quality of laboratory work, standardization and accuracy of laboratory reports.
